# Polar Superhelices in Ferroelectric Chiral Nanosprings

**DOI:** 10.1038/srep35199

**Published:** 2016-10-07

**Authors:** Takahiro Shimada, Le Van Lich, Koyo Nagano, Jian-Shan Wang, Jie Wang, Takayuki Kitamura

**Affiliations:** 1 Department of Mechanical Engineering and Science, Kyoto University, Nishikyo-ku, Kyoto 615-8540, Japan; 2 Tianjin Key Laboratory of Modern Engineering Mechanics, Department of Mechanics, Tianjin University, Tianjin 300072, China; 3 Department of Engineering Mechanics, School of Aeronautics and Astronautics, Zhejiang University, Hangzhou 310027, China

## Abstract

Topological objects of nontrivial spin or dipolar field textures, such as skyrmions, merons, and vortices, interacting with applied external fields in ferroic materials are of great scientific interest as an intriguing playground of unique physical phenomena and novel technological paradigms. The quest for new topological configurations of such swirling field textures has primarily been done for magnets with Dzyaloshinskii-Moriya interactions, while the absence of such intrinsic chiral interactions among electric dipoles left ferroelectrics aside in this quest. Here, we demonstrate that a helical polarization coiled into another helix, namely a polar superhelix, can be extrinsically stabilized in ferroelectric nanosprings. The interplay between dipolar interactions confined in the chiral geometry and the complex strain field of mixed bending and twisting induces the superhelical configuration of electric polarization. The geometrical structure of the polar superhelix gives rise to electric chiralities at two different length scales and the coexistence of three order parameters, i.e., polarization, toroidization, and hypertoroidization, both of which can be manipulated by homogeneous electric and/or mechanical fields. Our work therefore provides a new geometrical configuration of swirling dipolar fields, which offers the possibility of multiple order-parameters, and electromechanically controllable dipolar chiralities and associated electro-optical responses.

Topological objects of nontrivial field textures in condensed matter, such as vortices^1^,^2^,^3^, hedgehogs^4^,^5^,^6^, skyrmions^7^,^8^,^9^ and merons^10^,^11^,^12^, have attracted considerable attention and scientific interest as an intriguing avenue for both unique physical phenomena and their ability to be manipulated by external electric, magnetic, or mechanical fields, which offers a plethora of opportunities for novel functional nanodevice paradigms^13^,^14^. Numerous advances in physics and relevant technologies have been achieved due to the discovery of these topological states of matter, which has motivated researchers to further quest for new swirling or curling topologies of nontrivial field textures, especially in magnetic system due to the existence of intrinsic chiral interactions among spins, i.e., Dzyaloshinskii-Moriya (DM) interactions^15^. The chiral DM interaction is known to play an important role in stabilizing such topological spin textures as skyrmions^7^,^8^,^9^, which have attracted great interest since their discovery owing to their unconventional physical properties and potential technological applications. Although vortex or toroidal ordering and relevant flux-closure textures have already been found in ferroelectrics^2^,^16^,^17^,^18^,^19^,^20^,^21^, progress in discovering new topological configurations of swirling polarizations has been less active due to the absence of chiral interactions among electric dipoles.

Very recently, however, new swirling topological field textures of electrical polarization, such as electrical skyrmions^22^ and periodic vortex arrays^23^, have been discovered in complex ferroelectric systems such as cylindrical nanocomposites with (1–3) Newnham’s connectivity and multi-layered superlattices despite the absence of intrinsic chiral interactions. These pioneering studies into these objects imply that a key for such new swirling topologies in ferroelectrics lies on the interplay between discontinuity of dipolar interactions confined in a given geometry and the unique mechanical and electrostatic multi-fields intrinsic to the geometry, which lead to compensation of the large gradient energy intrinsic to curling or swirling polarizations through a counterbalance from the electrostatic and elastic energies. Swirling or curling polarization fields are therefore governed by the geometrical confinement of the system, which extrinsically provides a chiral character in the dipolar arrangement.

Inspired by the recent progress in discovering new swirling topological configuration of electrical polarization, here, we show that a helix of an electrical dipole coiled into another helix, namely a polar superhelix, can be extrinsically stabilized in ferroelectric nanosrpings using phase-field modelling based on the Ginzburg-Landau theory. The superhelical geometry of electric polarization realizes the coexistence of three different order parameters, polarization, toroidization, and hypertoroidization, as well as electric chiralities at two different length scales in the ferroelectric nanosprings. Both of these phenomena can be manipulated by an applied electric field and/or mechanical load. The interplay between dipolar interactions confined in the chiral geometry and the complex strain field of the mixed bending and twisting stabilizes the superhelical configuration of the electric polarization. Our result therefore provides a new class of swirling geometrical fields of electric dipoles, which offers the possibility of manipulating multiple order parameters and electric chiralities, and could be particularly useful for the development of nano-electromechanical and electro-optical devices.

## Results

### Superhelical polarization in ferroelectric nanosprings

Chirality, which introduces handedness to a system, breaks the inversion and mirror symmetries of the system, which are absent in the conventional nanostructures with simple shapes, i.e., thin films, nanowires, and nanodots. Such a unique and intriguing structural characteristic provides an ideal playground for the quest of new swirling topological field textures of electrical polarization. We focus on a PbTiO_3_ ferroelectric nanospring in the present study, which inherently possesses structural chirality. Based on recent fabrication advances^24^,^25^,^26^, we consider the configuration of the PbTiO_3_ ferroelectric nanospring with outer diameter *D* = 100 nm, wire diameter *d* = 25 nm, and helical angle ***α*** = 20°, as shown in Supplementary Figure S1. The formation of spontaneous polarization in a ferroelectric nanospring specimen in the absence of external fields at room temperature is carefully tested using real-space phase-field techniques based on the Ginzburg-Landau theory, which explicitly includes the depolarization effect and nontrivial electro-elastic coupling (see the Method section) and successfully reproduces and explains the recently observed polar vortex arrays^23^.

**Figure 1:**
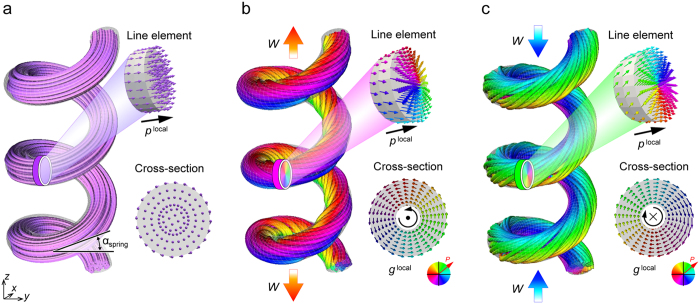
Polarization configuration in ferroelectric nanosprings. (**a**) Helix polarization structure in ferroelectric nanosprings in the absence of external fields. The line element and cross-section of the ferroelectric nanospring show a detailed polarization distribution in a local area. (**b**) Right-handed (RH) polar superhelix in the ferroelectric nanospring under tensile load of ***W*** = 240 μN. (**c**) Left-handed (LH) polar superhelix in the ferroelectric nanospring under the compressive load. The contour colour indicates the direction of the polarization vectors in the cross-section of the nanospring.

Figure 1(a) shows the spontaneous polarization distribution in the ferroelectric nanospring in the thermodynamic equilibrium state in the absence of mechanical load or external electric field. Polarization vectors continuously flows along the axial direction of the nanospring, keeping a head-to-tail arrangement. Such a continuous flow causes a significant decrease in the overall electrostatic interaction energy through elimination or reduction of the depolarization fields at the surfaces, and is thereby energetically favourable. A magnification view of a line element and cross-section of the nanospring show that the polarization almost uniformly aligns in the axial direction of the nanospring. Thus, the polarizations form a helical polarization configuration in nature that is driven by the unique chirality of the nanospring shape. The characteristics of the continuous flow are consistent with those observed in preceding studies of ferroelectric nanowires^27^, nanorings^28^, and nano-metamaterials^21^, where the polarization pattern is significantly governed by the outer shape of the nanostructure. In addition, a recent experiment has observed a similar global magnetization helix in a magnetic nanospiral^29^. These consistencies validate the reliability of the present phase-field simulations, and indicate that a global helical polarization pattern is characteristic of ferroelectric nanosprings.

Figure 1(b,c) show the polarization distribution in the ferroelectric nanospring under tension and compression, respectively. The local polarization configuration in the line element shown in Fig. 1(b) features polarizations along the axial direction of the nanospring, co-occurring with a counter-clockwise vortex structure of the cross-sectional polarization field, producing an in-plane pattern whereby the strength of the depolarization field is reduced^30^. The local polarization configuration in the line element, thus, exhibits a microscopic helical structure. Interestingly, the microscopic helical structure twists around the axial direction of the nanospring to maintain the continuous flow through the entire structure, giving rise to another helical configuration occurring simultaneously at a different length scale, i.e., a global helical configuration. The simultaneous occurrence of helical structures at two different length scales within a single ferroelectric nanospring forms a double-twisted or hierarchical helix polarization; so called a superhelix. Such a superhelical polarization has never been reported for ferroelectric or magnetic domain configurations. Thus, the polar superhelix is an unusual domain configuration emerging in ferroelectric nanosprings. Figure 1(b) shows a right-handed (RH) superhelix structure. Such a polar superhelix is also observed under compression (Fig. 1(c)). However, a characteristic that is distinct from the tension case is a clockwise twisting of the local helix polarization, which wraps around the axial direction of the nanospring, forming a left-handed (LH) superhelix polarization structure. Therefore, the unconventional polar superhelices can be formed in ferroelectric nanospring under mechanical field, where the chirality of the superhelix can be controlled by the loading state.

**Figure 2:**
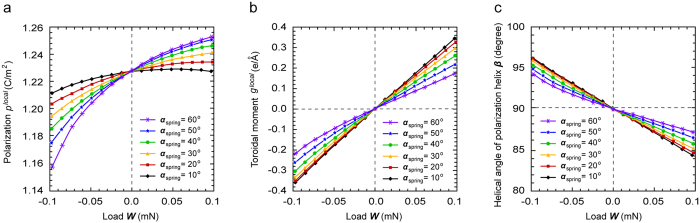
Change in microscopic (local) characteristic properties of a polarization helix under mechanical field. (**a**) Local polarization ***p***^***local***^, (**b**) local toroidal moment ***g***^***local***^, and (**c**) helical angle of polarization helix ***β***^***pol***^ as functions of applied mechanical load.

To understand the characteristics of ferroelectric nanosprings with polar superhelices, we first probe the properties from a microscopic view point. Figure 2(a,b) shows the local polarization ***p***^local^ and local toroidal (i.e., poloidal) moment ***g***^local^ as a function of the mechanical load ***W***, respectively. The local polarization ***p***^local^ is defined as the volume averaged polarization in the line element with the direction along the longitudinal direction of the line element, and is expressed by:


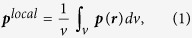


where ***p***(***r***) is the polarization at the position ***r*** and ***v*** is the volume of line element. The local toroidal moment ***g***^local^, which characterizes the ferrotoroidic properties of the local helical polarization formed in the line element of nanospring, is expressed as^2^


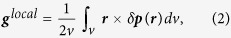


where *δ**p***(***r***) is the transverse polarization (*δ**p***(***r***) = ***p***(***r***)−***p***^local^) at the position ***r***. In addition, we also investigate the effect of the helical angle of the nanospring ***α*** on the mechanical response of the polarization, considering nanosprings with different helical angles (***α*** = 10°, 20°, 30°, 40°, 50° and 60°). Figure 2(a) shows that ***p***^local^ gradually increases with an increase in the mechanical load. ***p***^local^ becomes more sensitive to the mechanical load when ***α*** increases. A similar tendency is observed for ***g***^local^ in the Fig. 2(b), in which ***g***^local^ linearly increases with an increase in the mechanical load. However, ***g***^local^ becomes less sensitive to the mechanical load when ***α*** increases. These results can be attributed to the fact that the applied tensile (or compressive) load to the nanospring induces tension (or compression) and torsion components in the line element^31^, which cause an increase (or decrease) in the axial component and circumferential component of the polarization, respectively. In addition, a change in ***α*** gives rise to a simultaneous change in the magnitude of the tension/compression and torsion components in the line element^31^, resulting in a change in the sensitivity of ***p***^local^ and ***g***^local^ to the mechanical load. Therefore, both the local polarization and local toroidal moment are sensitive to the mechanical load; the larger the mechanical load, the larger the local polarization and local toroidal moment.

Since the microscopic polarization helix structure intrinsically coexists with the local polarization and local toroidal moment, we introduce here a helical angle of polarization ***β***, in order to characterize this coexistence. ***β*** represents the chirality and winding density of the microscopic helical polarization. The greater ***β*** is, the smaller the winding density. ***β*** is expressed by:


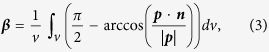


where ***n*** is the unit vector in the axial direction of the cross-section. Figure 2(c) shows ***β*** as a function of mechanical load ***W*** with different ***α***. In the absence of a mechanical load, ***β*** is equal to 90°, which corresponds to a uniform polarization along the axial direction of the nanospring, where only ***p***^local^ occurs. The helical angle of polarization ***β*** decreases gradually from 90°, and thereby, its winding density increases with an increase in tensile load. ***β*** < 90° corresponds to the microscopic RH helical polarization, where both local polarization and counter-clock wise vortex occur. On the other hand, ***β*** increases gradually from 90° with an increase in the compressive load, giving rise to a microscopic LH helical polarization. The winding density becomes larger with an increase in compressive load. In addition, ***β*** is less sensitive to the mechanical load when ***α*** increases. Therefore, the mechanical load not only induces a microscopic polarization helix, but can also alter the polarization helix configuration, i.e., the chirality and helical angle ***β***, through controlling both ***p***^local^ and ***g***^local^.

### Multiple order parameters

**Figure 3:**
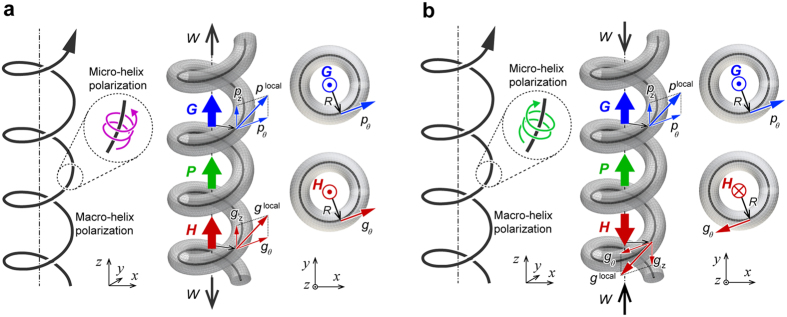
Schematic illustration of order parameters constituting a polar superhelices in ferroelectric nanosprings. (**a**) Under a tensile load and (**b**) under a compressive load. The large blue, green, and red arrows indicate vectors of the global toroidal moment ***G***, global polarization ***P***, and hypertoroidal moment ***H***, respectively.

Given the intriguing features of the polarization superhelix structure in ferroelectric nanosprings, we consider the macroscopic ferroelectric properties to formulate a comprehensive picture of the interrelations among the nanospring structure, polarization patterns, and global behaviour in the ferroelectric nanospring. The ferroelectric domain structure is commonly characterized by a sole order parameter due to its simple configuration, exemplified by the overall polarization for the single and multiple domain states in bulk ferroelectrics or toroidization for the vortex state in a nanodot. Since both the polarization configuration characteristics appear in a ferroelectric nanospring, Fig. 3 schematically depicts the average polarization ***P*** and global toroidal moment ***G***, which are quantitatively described as follow:


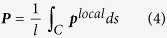



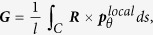


where 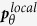 indicates the circumference component of ***p***^local^, ***R*** is the spatial vector from the central axis of the nanospring, and *l* is the total length of the curve of the wire *C*. ***P*** represents the average polarization in the entire nanospring, while ***G*** represents the strength and circulation of the polarization. Although the combination of the ***P*** and ***G*** order parameters can describe well the global helix polarization configuration, as shown in Fig. 1(a), it cannot fully represent the complex superhelix polarization structure, where two different length scales of helix polarization occur simultaneously in the ferroelectric nanospring under a mechanical load. Therefore, an additional order parameter is necessary for such a complex polarization pattern. We can define another order parameter, the hypertoroidal moment ***H***, which involves a cross product of local toroidizations with the vectors locating their positions, in order to characterize the curl of the ferroelectric toroidization. ***H*** is schematically represented in Fig. 3, and its physical quantity is described as follows:


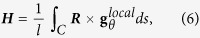


where 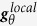 indicates the circumference component of ***g***^local^. The inherent complexity in the superhelix polarization configuration manifests itself by the coexistence of several characteristics associated with ferroelectric order parameters and thereby requires a conjugation of at least three order parameters described above. A demonstration that the conjugation of the three order parameters represents entirely the complex superhelix polarization is discussed below. Therefore, the polar superhelices in ferroelectric nanosrpings bring about multi-order-parameters of the (averaged) polarization, toroidization, and hypertoroidic moment.

**Figure 4:**
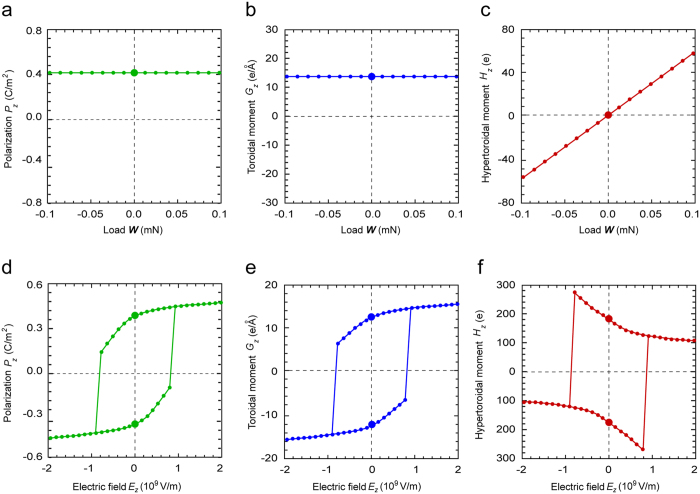
Switching of order parameters by applied mechanical and/or electric field. (**a**–**c**) Change of macroscopic (global) characteristic properties of a polarization superhelix under mechanical and electric fields. (**a**) Global polarization, (**b**) global toroidal moment, and (**c**) hypertoroidal moment as functions of applied mechanical load. (**d**–**f**) Hysteresis loops of the switchings of (**d**) the global polarization, (**e**) global toroidal moment and (**f**) hypertoroidal moment under an electric field.

To investigate the susceptibility of the order parameters in the ferroelectric nanospring to external fields, their responses under mechanical and electrical fields are considered. Figure 4(a–c) shows ***P***, ***G*** and ***H***, respectively, as functions of the mechanical load ***W***. The average polarization and global toroidal moment is almost constant under mechanical load, while the hypertoroidal moment linearly increases with an increase in the load and vice versa. The magnitude of ***H*** is positive under tension, but is negative under compression. This demonstrates that the hypertoroidal moment in the ferroelectric nanospring can be switched by a mechanical field, providing a way to control such a hypertoroidal moment. Figure 4(d–f) shows the responses of ***P***, ***G*** and ***H***, respectively, to a homogeneous electric field in the *z* direction, *E*_*z*_. All the order parameters exhibit a hysteresis loop, in which an irreversible switching between positive and negative values takes place. Therefore, the polar superhelices in the ferroelectric nanospring carry three distinct order parameters that can be controlled by both mechanical and electrical fields, suggesting a promising potential for multiple control of the polar superhelices.

### Electric chiralities in polar superhelices

**Figure 5:**
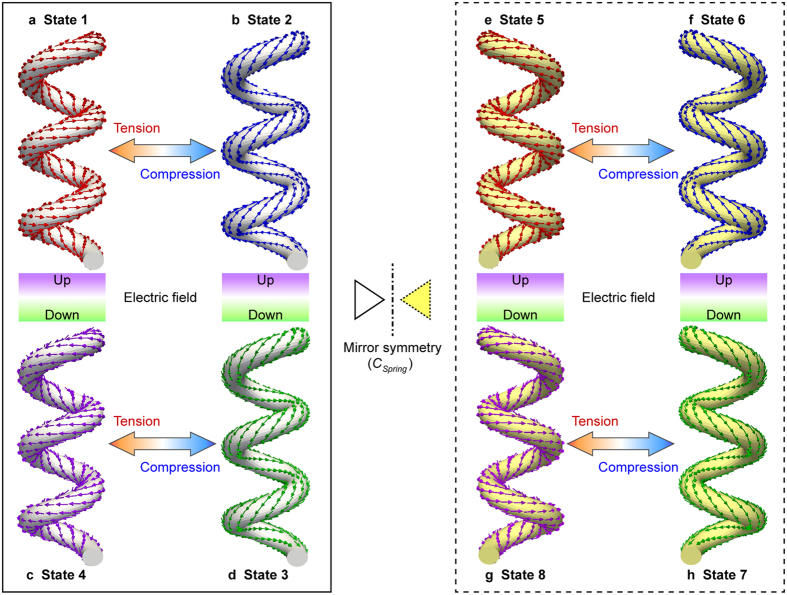
Electric chiralities in ferroelectric nanosprings. Possible states with different chiralities for polar superhelices in ferroelectric chiral nanosprings. Arrows indicate possible mechanical/electric controls of the states.

Table 1 summarizes the obtainable states of the polar superhelices and the corresponding conditions of the three order parameters. From this we can identify the symmetry of the superhelical structure. The possible states are defined based on a combination of the three order parameters. For instance, state 1 is characterized by the macroscopic order parameters of positive ***P***_*z*_, positive ***G***_*z*_, and positive ***H***_*z*_, which is described here as (+/+/+). Visualizations of all the states and the routes to transform from one state to another are depicted in Fig. 5. When the mechanical load changes from tension to compression, the microscopic toroidal moment switches from positive to negative, resulting in the exclusive switching of the hypertoroidal moment without any change in the other parameters. Thereby, the state (+/+/−), i.e., state 2, can be achieved (Fig. 5(b)). When an external electric field is applied to state 1 or state 2, all of the order parameters are switched, resulting in states 4 (−/−/−) or 3 (−/−/+), respectively. States 3 and 4 are shown in Fig. 5(d,c), respectively. In addition, when a static rotation-reversal symmetry^32^ is applied to the chirality of a nanospring (*C*_*spring*_), which transforms a RH nanospring to a LH nanospring, four more states are obtained. Under rotation-reversal symmetry, the polarization superhelices can be changed to the opposite ***G***_*z*_ and ***H***_*z*_ with respect to the states 1–4, as shown in the states 5–8 ((+/−/−), (+/−/+), (−/+/−), and (−/+/+)). This is because *C*_*spring*_ constrains the combination of ***P***_*z*_ and ***G***_*z*_: (***P***_*z*_, ***G***_*z*_) achieves (+,+) or (−,−) in the RH chirality, while (***P***_*z*_, ***G***_*z*_) is (+,−) or (−,+) in the LH chirality. Thus, the chirality of the nanospring *C*_*spring*_ can produce independent and additional varieties of polar superhelical states with unfettered combinations of the order parameters ***P***_*z*_, ***G***_*z*_ and ***H***_*z*_. States 5–8 can also be switched from one to another by a mechanical or electric field. Therefore, eight distinct states, which are fully described by a combination of the three order parameters, emerges equivalently in the polar superhelices and their transformation from one to another can be obtained through a mechanical load, electric field, and the chirality of the nanospring.

## Discussion

The superhelical configuration of electric polarization emerges as a consequence of an interplay between dipolar interactions confined in the chiral geometry and the complex strain field of the mixed bending and twisting. The unique topology of nanosprings, in addition to the size effects at nanoscales, induces a chiral polarization configuration, appearing as a ground state, providing a prerequisite for extrinsic chiral interactions to occur in the ferroelectric nanospring. On the other hand, even if a uniaxial mechanical field is imposed on the nanospring, a complex strain field of mixed bending and twisting arises due to the structural characteristics of the nanospring. The mixing of the bending and twisting, the corresponding stress distributions of which are provided in Supplementary Figure S2, provides an extra elastic energy into the system that leads to a compensation of the large gradient energy intrinsic to the curling or swirling polarizations through a nontrivial electromechanical cross-coupling to counterbalance the electrostatic and elastic energies (Supplementary Figures S3 and S4). The opposite tension (bending) and shear (twisting) stress states induced by the uniaxial tension and compression, which provides an underlying mechanism for stabilizing the RH and LH superhelix polarization configurations in tensile and compressed ferroelectric nanosprings. Therefore, the interplay between dipolar interactions confined in the chiral geometry and the complex strain field of the mixed bending and twisting plays a significant role in the emergence of extrinsic chiral interactions in ferroelectrics that help to stabilize the novel superhelix polarization structure.

On the other hand, inhomogeneous stress or strain distribution in the ferroelectric nanospring under axial mechanical load, which is shown in Supplementary Figure S2, exhibits a large strain gradient that leads to flexoelectricity. The flexoelectric effect contributes to enhance the coupling between polarization and strain^33^. Since the coupling between polarization and strain is one of the source for the superhelical configuration stabilization, the flexoelectric effect may further enhance the magnitude of both toroidal and hypertoroidal moments. The topology of superhelical polarization configuration does not change in general due to the dominant of chiral shape effect. Therefore, the flexoelectricity in ferroelectric nanospring can enhance both the toroidicity and hypertoroidicity. However, a quantitative consideration for the influence of flexoelectric effect on magnitude of toroidal and hypertoroidal moments is kept for future work.

The nanospring structure, which inherently possesses strong chirality, offers a new twist to the emerging field of electro-optical properties^34^. Such chiral optical materials mix electrical and magnetic responses so that the magnetic/electric dipoles are induced by the electric/magnetic components of the light field. In addition, recently promising work has been carried out on potential optical application of the polarization vortices or helices^23^,^35^,^36^. Theoretical contributions proposed in recent works^35^,^36^ show that electrotoroidic (or ferrotoroidic) systems with electrical polarization perpendicular to the vortex plane are chiral, and therefore, naturally gyrotropic, i.e., they spontaneously possess optical activity. The optical activity is characterized by the gyrotropy tensor^35^,^36^ that depend on toroidal and hypertoroidal moments. Ferroelectric nanospring inherently achieves strong gyrotropy not only due to its chiral outer shape, but also from the substructure of the superhelix polarization, which are denoted as *C*_*spring*_ and *C*_*polar*_, respectively, as listed in Table 1. For each static *C*_*spring*_, the *C*_*polar*_, which is associated with the three order parameters, is controllable by external fields. Because of the complex geometrical chirality of superhelical polarization configuration, it is expected to achieve enhanced gyrotropy coefficient, and hence, strong optical activity in the ferroelectric nanospring with superhelical polarization, offering exciting prospects for devices such as nanoscale optical circulators. This also provides a promising playground for the complicated mutual interactions among the helical structure, superhelix polarization configuration, and components of the light field. These mutual interactions indicate a new class of electro-optical materials, where the ferroelectric nanospring acts as a source controllable gyrotropy of the polarization superhelix can bring about an unusual type of nonlinear response^37^.

In summary, we have demonstrated that a novel superhelix polarization configuration can be extrinsically stabilized in ferroelectric nanosprings. The geometrical structure of polar superhelices induces electric chiralities at two different length scales and the coexistence of three order parameters, i.e., polarization, toroidization, and hypertoroidization, and both of these phenomena can be switched by homogeneous electric and/or mechanical fields. This work has revealed the underlying mechanism of the interplay between dipolar interactions confined in the chiral geometry and a complex strain field of mixed bending and twisting, which electromechanically stabilizes the superhelical configuration of the electric polarization. This result will pave the way to control their behaviour and interactions, leading to the capability to design desired properties and functionalities that could be particularly useful for the development of nano-electromechanical and electro-optical devices.

## Method

### Phase-field modelling

In the phase-field modelling of ferroelectric nanosprings, the evolution of the polarization and thermodynamic equilibrium was calculated by solving the time-dependent Ginzburg-Landau (TDGL) equations:


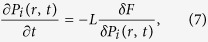


where *t* denotes time, *L* is a kinetic coefficient, *r* = (*x*, *y*, *z*) denotes the spatial vector, and *δF*/*δP*_*i*_(***r***, *t*) denotes the thermodynamic driving force for the evolution of the polarization. The total free energy of the ferroelectric system *F*, includes the Landau energy, elastic energy, coupling energy, gradient energy, and electrostatic energy, and can be described as:


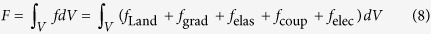


Detailed expressions of the energy densities are presented in some depth in our previous studies^20^,^21^,^38^ and in the Supplementary Materials. A backward Euler scheme and Newton iteration method were used for the time integration and nonlinear iteration, respectively.

Periodic boundary conditions are applied to the simulation model in the length direction of the nanospring. To investigate the effect of tensile and compressive load, a small increment of load, Δ***W***, is applied stepwise in the axial direction of the nanospring. It should be noted that the mechanical load of ***W*** is equivalent to a set of tension, shear, bending, and torsion on the cross-section of the nanospring, which can be expressed as follows^31^:


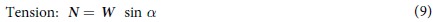



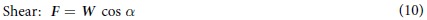



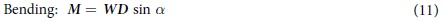



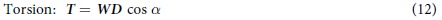


where ***α*** is the helical angle of the nanospring and ***D*** is the outer diameter of the nanospring. Thus, the set of equivalent forces to the mechanical load ***W*** is applied stepwise to the upper and lower planes of the simulation model, which realize an identical mechanical state with a periodic ferroelectric nanospring. The values of the material coefficients for PbTiO_3_ employed in this study are the same as in refs 20, 21 and 38.

## Additional Information

**How to cite this article**: Shimada, T. *et al*. Polar Superhelices in Ferroelectric Chiral Nanosprings. *Sci. Rep*. **6**, 35199; doi: 10.1038/srep35199 (2016).

## Supplementary Material

Supplementary Information

## Figures and Tables

**Table 1:** Possible states of polar superhelices in ferroelectric nanosprings.

	State 1	State 2	State 3	State 4	State 5	State 6	State 7	State 8
*p*^*local*^	+	+	−	−	+	+	−	−
*g*^*local*^	+	−	+	−	+	−	+	−
*P*_*z*_	+	+	−	−	+	+	−	−
*G*_*z*_	+	+	−	−	−	−	+	+
*H*_*z*_	+	−	+	−	−	+	−	+
*C*_*polar*_	RH	LH	LH	RH	RH	LH	LH	RH
*C*_*spring*_	RH				LH			
