# Supplementary material for: Polar Superhelices in Ferroelectric Chiral Nanosprings
Source: Sci Rep. 2016 Oct 7;6:35199. doi: 10.1038/srep35199 (PMC5054384; doi:10.1038/srep35199)
Supplement: Supplementary Information [file srep35199-s1.pdf]

Supplementary Material for

Polar Superhelices in Ferroelectric Chiral Nanosprings

Takahiro Shimada,[1,*] Le Van Lich,[1,*] Koyo Nagano,[1] Jian-Shan Wang,[2] Jie Wang,[3] and Takayuki Kitamura[1]

[1] *Department of Mechanical Engineering and Science, Kyoto University, Nishikyo-ku, Kyoto 615-8540, Japan*

[2] *Tianjin Key Laboratory of Modern Engineering Mechanics, Department of Mechanics, Tianjin University, Tianjin 300072, China*

[3] *Department of Engineering Mechanics, School of Aeronautics and Astronautics, Zhejiang University, Hangzhou 310027, China*

Supplementary Figures

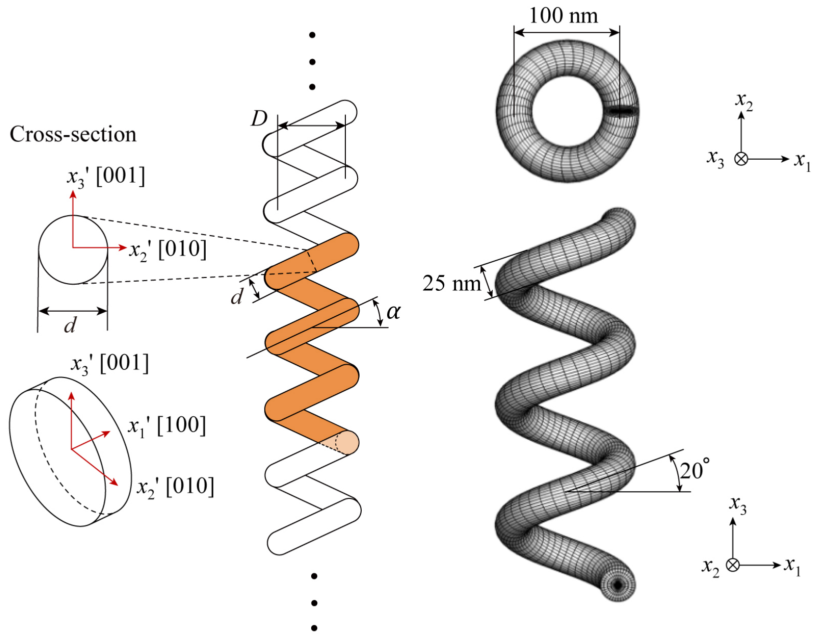

Figure S1. Ferroelectric nanospring for phase-field modeling.

*These authors contributed equally to this work. Correspondence and requests for materials should be addressed to T.S. (email: shimada@me.kyoto-u.ac.jp).

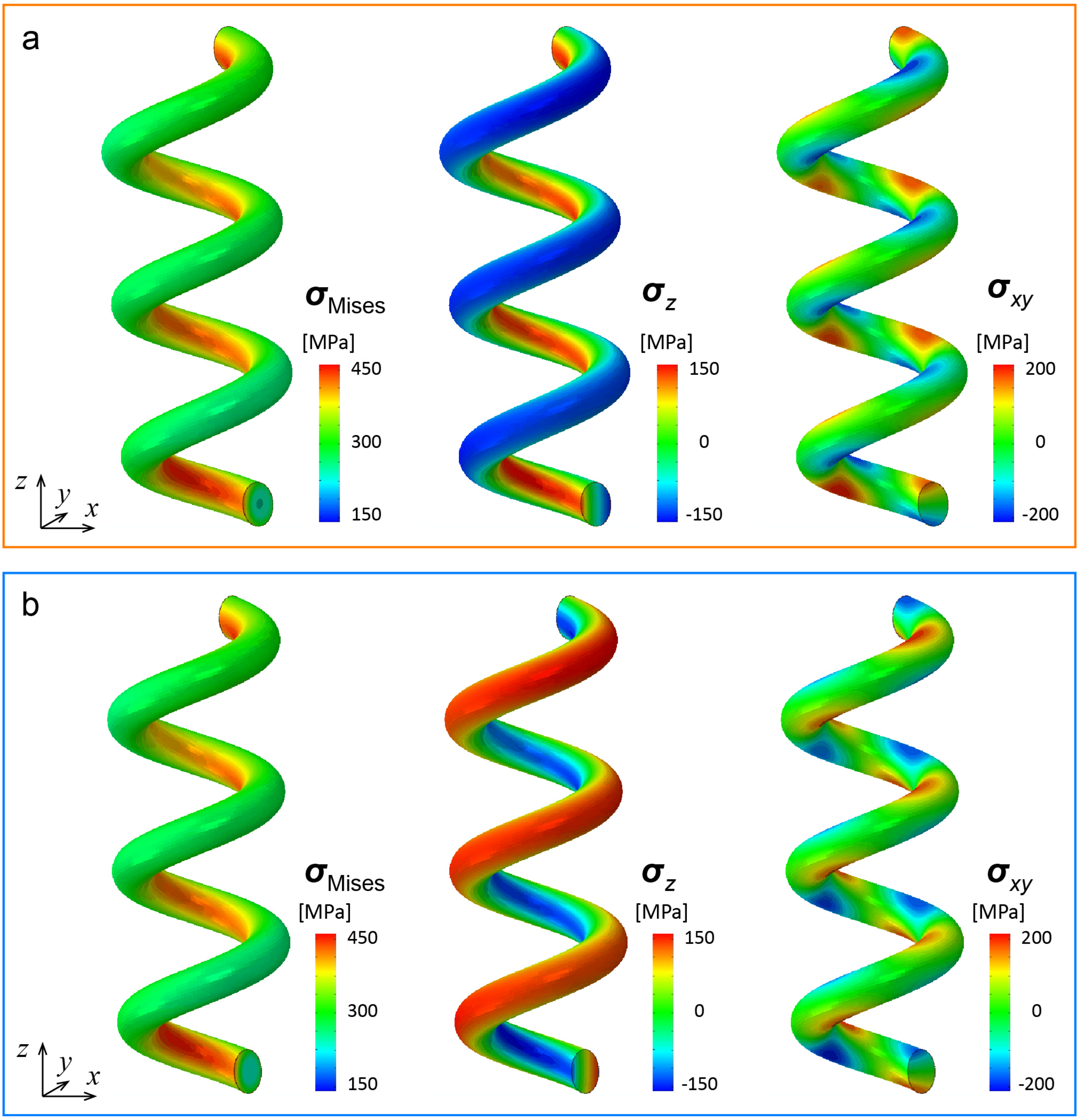

Figure S2. Stress distribution in the ferroelectric nanospring under (a) external tensile load and (b) external compressive load.

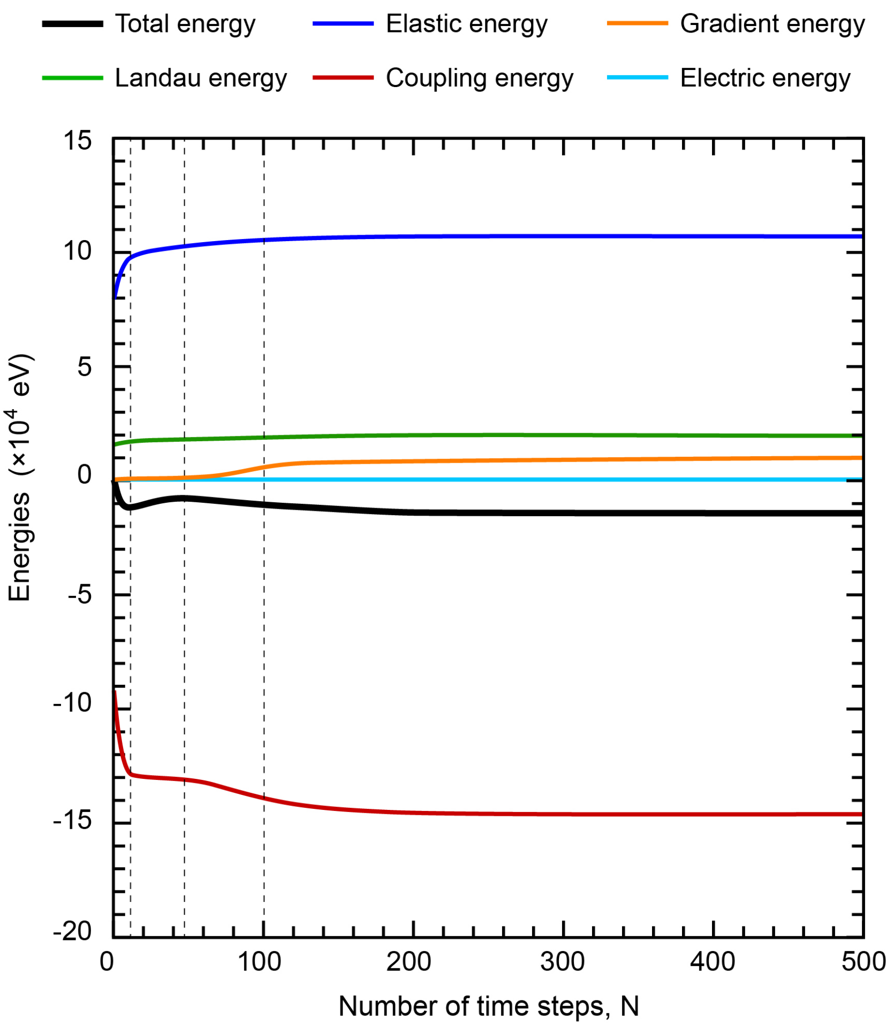

Figure S3. Temporal evolution of the various energy contributions during the formation of superhelical polarization configuration. The total energy decreases during the evolution. Only the coupling energy is observed to decrease, which causes the decrease of the total free energy, while the other energy component remain constant or increase. This indicates that the electro-elastic coupling energy is driving force for the formation of superhelical polarization configuration in the ferroelectric nanospring under mechanical excitation.

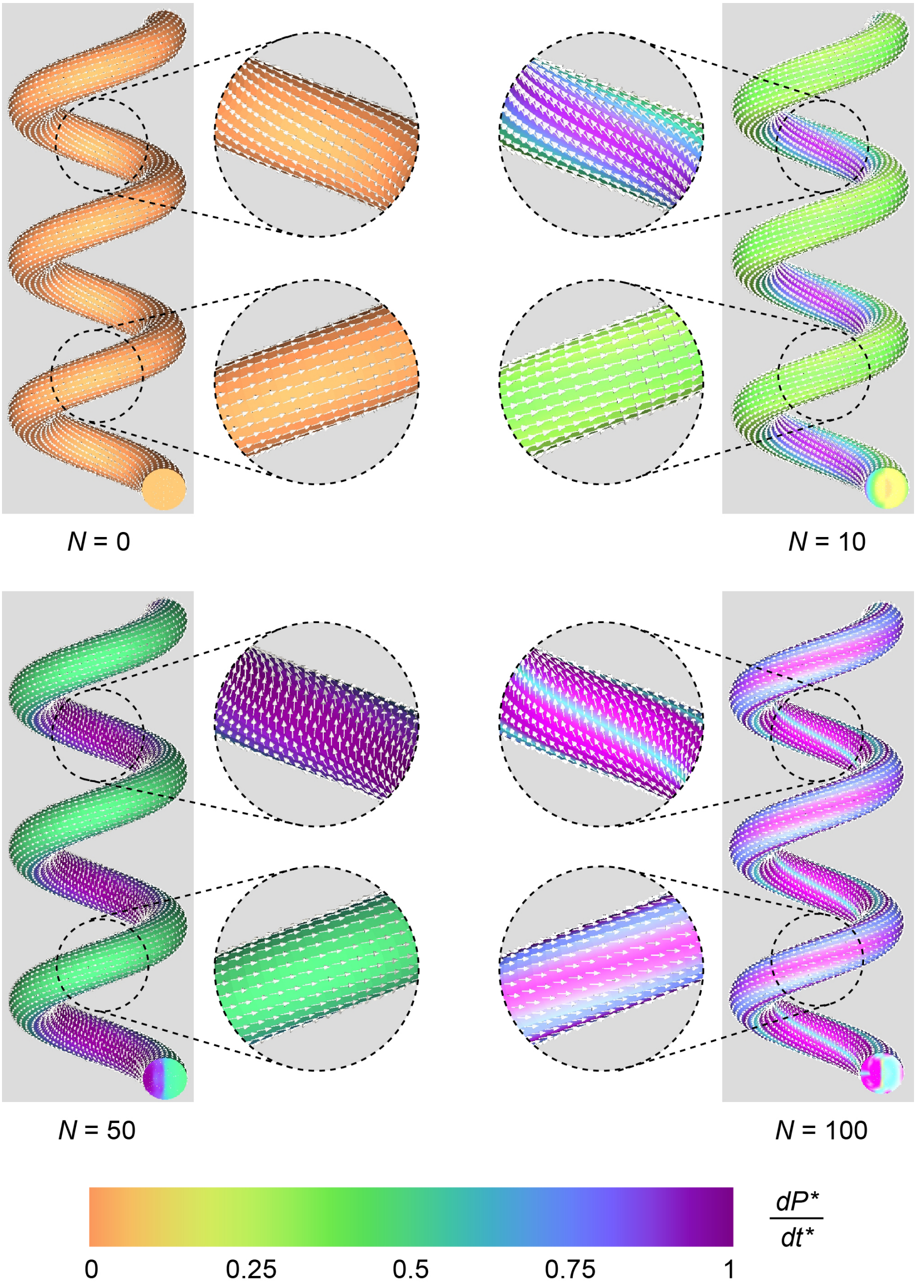

Figure S4. Change of polarization in the ferroelectric nanospring during the formation of superhelical polarization configuration. First, the polarization change quickly at the inner side of the ferroelectric nanospring (N=10 and 50), then propagates to the outer side (N=100).

Phase-field modeling.

In the phase-field model of ferroelectric materials, polarization vector, $\boldsymbol{P} = (P_1, P_2, P_3)$, is taken as the order parameter to describe free energies of the ferroelectric system. The total free energy of the ferroelectric system, F, can be described by [1]:

$$F = \int_V f dV = \int_V \left(f_{Land} + f_{elas} + f_{coup} + f_{grad} + f_{elec} \right) \, dV \tag{S1}$$

where f_{Land}, f_{elas}, f_{coup}, f_{grad}, and f_{elec} denote the Landau energy density, elastic energy density, coupling energy density, gradient energy density, and electrostatic energy density, respectively. V is the entire volume of the ferroelectric system.

The Landau energy density is expressed by a six-order polynomial of the spontaneous polarization as [2]:

$$\begin{aligned} f_{Land} &= \alpha_1\left(P_1^2 + P_2^2 + P_3^2\right) + \alpha_{11}\left(P_1^4 + P_2^4 + P_3^4\right) \\ &+ \alpha_{12}\left(P_1^2 P_2^2 + P_2^2 P_3^2 + P_3^2 P_1^2\right) + \alpha_{111}\left(P_1^6 + P_2^6 + P_3^6\right) \\ &+ \alpha_{112}\left[P_1^4\left(P_2^2 + P_3^2\right) + P_2^4\left(P_1^2 + P_3^2\right) + P_3^4\left(P_1^2 + P_2^2\right)\right] \\ &+ \alpha_{123} P_1^2 P_2^2 P_3^2 \end{aligned} \tag{S2}$$

where $\alpha_1 = (T - T_0) / 2\kappa_0 C_0$ is the dielectric stiffness, α_{11}, α_{12}, α_{111}, α_{112}, and α_{123} are higher order-stiffness coefficients, T and T_0 denote the temperature and the Curie-Weiss temperature, respectively, C_0 denotes the Curie constant, and κ_0 denotes the dielectric constant of vacuum. The strain energy density is given by:

$$f_{elas} = \frac{1}{2} c_{11}\left(\varepsilon_{11}^2 + \varepsilon_{22}^2 + \varepsilon_{33}^2\right) + c_{12}\left(\varepsilon_{11}\varepsilon_{22} + \varepsilon_{22}\varepsilon_{33} + \varepsilon_{33}\varepsilon_{11}\right) + 2c_{44}\left(\varepsilon_{12}^2 + \varepsilon_{23}^2 + \varepsilon_{31}^2\right), \tag{S3}$$

where c_{11}, c_{12}, and c_{44} are the elastic constants. The coupling energy density is given by:

$$\begin{aligned} f_{coup} &= -q_{11}\left(\varepsilon_{11} P_1^2 + \varepsilon_{22} P_2^2 + \varepsilon_{33} P_3^2\right) - q_{12}\left[\varepsilon_{11}\left(P_2^2 + P_3^2\right) + \varepsilon_{22}\left(P_3^2 + P_1^2\right) + \varepsilon_{33}\left(P_1^2 + P_2^2\right)\right] \\ &- 2q_{44}\left(\varepsilon_{11} P_1 P_2 + \varepsilon_{13} P_1 P_3 + \varepsilon_{23} P_2 P_3\right) \end{aligned} \tag{S4}$$

where q_{11}, q_{12}, and q_{44} are electrostrictive coefficients. The gradient energy density is given by:

$$f_{grad} = \frac{1}{2} G_{11}\left(P_{1,1}^2 + P_{2,2}^2 + P_{3,3}^2\right) + G_{12}\left(P_{1,1} P_{2,2} + P_{2,2} P_{3,3} + P_{3,3} P_{1,1}\right) \tag{S5}$$

$$+\frac{1}{2}G_{44}\left[(P_{1,2}+P_{2,1})^2+(P_{2,3}+P_{3,2})^2+(P_{1,3}+P_{3,1})^2\right]$$

$$+\frac{1}{2}G'_{44}\left[(P_{1,2}-P_{2,1})^2+(P_{2,3}-P_{3,2})^2+(P_{1,3}-P_{3,1})^2\right]$$

where G_{11}, G_{12}, G_{44}, and G'_{44} are the gradient coefficients. The gradient energy is the penalty for the spatially inhomogeneous polarization. The electrostatic energy density, which is obtained through Legendre transformation, is given as:

$$f_{elec}=-\frac{1}{2}\kappa_0\left(E_1^2+E_2^2+E_3^2\right)-E_1P_1-E_2P_2-E_3P_3. \quad \text{(S6)}$$

The temporal evolution for polarization or domain structure is calculated by the time-dependent Ginzburg-Landau equation:

$$\frac{\partial P_i(\boldsymbol{r},t)}{\partial t}=-L\frac{\delta F}{\delta P_i(\boldsymbol{r},t)}, \quad \text{(S7)}$$

where t represents time, L is the kinetic coefficient related to the domain mobility, $\delta F/\delta P_i(\boldsymbol{r}, t)$ denotes the thermodynamic driving force for polarization evolution, and $\boldsymbol{r}$ is the spatial vector. In addition to the time-dependent Ginzburg-Landau equation, the following mechanical equilibrium equation

$$\frac{\partial}{\partial x_j}\left(\frac{\partial f}{\partial \varepsilon_{ij}}\right)=0 \quad \text{(S8)}$$

and Maxwell's (or Gauss) equation

$$\frac{\partial}{\partial x_i}\left(-\frac{\partial f}{\partial E_i}\right)=0 \quad \text{(S9)}$$

must be satisfied for charge and body force free ferroelectric materials simultaneously.

Using the variation or principal of virtual work, the governing Equations (7)-(9) are expressed in the integral form (or weak form) as [3]:

$$\int_V\left\{\frac{\partial f}{\partial \varepsilon_{ij}}\delta\varepsilon_{ij}+\frac{\partial f}{\partial E_i}\delta E_i+\frac{1}{L}\frac{\partial P_i}{\partial t}\delta P_i+\frac{\partial f}{\partial P_i}\delta P_i-\left(\frac{\partial f}{\partial P_{i,j}}\right)\delta P_{i,j}\right\}dv$$

$$=\int_S\{t_i\delta u_i-wd\varphi+\pi_i\delta P_i\}dA, \quad \text{(S10)}$$

where t_i is the surface traction, w denotes surface charge, and $\pi_i = \frac{\partial f}{\partial P_{i,j}}n_j$ represents the surface

gradient flux. A nonlinear finite element method [1] is employed to solve Eq. (S10), which is suitable for the arbitrary geometry of ferroelectric nano-mesomaterials.

References

[1] Wang, J. & Kamlah, M. Three dimensional finite element modeling of polarization switching in a ferroelectric single domain with an impermeable notch. *Smart Mater. Struct.* **18**, 104008 (2009).

[2] Wang, J. Switching mechanism of polarization vortex in single-crystal ferroelectric nanodots. *Appl. Phys. Lett.* **97**, 192901 (2010).

[3] Wang, J., Kamlah, M., Zhang, T. Y., Li, Y. & Chen, L. Q. Size dependent polarization distribution in ferroelectric nanostructures: Phase field simulations. *Appl. Phys. Lett.* **92**, 162905 (2008).
